# Ethyl 2-(bromo­meth­yl)-5-meth­oxy-1-phenyl­sulfonyl-1*H*-indole-3-carboxyl­ate

**DOI:** 10.1107/S1600536808007319

**Published:** 2008-03-20

**Authors:** G. Chakkaravarthi, Radhakrishnan Sureshbabu, A. K. Mohanakrishnan, V. Manivannan

**Affiliations:** a Department of Physics, CPCL Polytechnic College, Chennai 600 068, India; b Department of Organic Chemistry, University of Madras, Guindy Campus, Chennai 600 025, India; c Department of Physics, Presidency College, Chennai 600 005, India

## Abstract

In the title compound, C_19_H_18_BrNO_5_S, the plane of the phenyl ring forms a dihedral angle of 76.99 (6)° with the indole ring system. The Br atom is disordered over two positions, with site-occupancy factors of 0.833 (14) and 0.167 (14). The mol­ecular structure is stabilized by weak intra­molecular C—H⋯O inter­actions and the crystal packing is stabilized by weak inter­molecular C—H⋯O inter­actions.

## Related literature

For biological activity, see: Nieto *et al.* (2005 ▶); Yang *et al.* (2002 ▶). For the structures of closely related compounds, see: Chakkaravarthi *et al.* (2007 ▶, 2008 ▶).
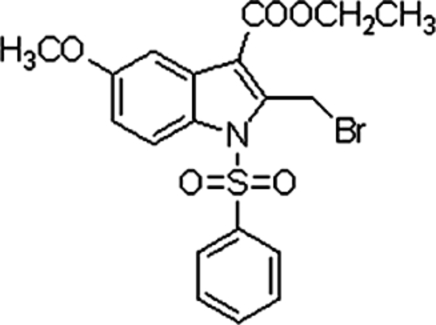


## Experimental

### 

#### Crystal data

C_19_H_18_BrNO_5_S
*M* *_r_* = 452.31
Triclinic, 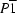

*a* = 8.9988 (3) Å
*b* = 9.2343 (2) Å
*c* = 11.6068 (3) Å
α = 82.524 (1)°
β = 87.666 (2)°
γ = 84.942 (3)°
*V* = 952.16 (5) Å^3^
*Z* = 2
Mo *K*α radiation
μ = 2.30 mm^−1^
*T* = 295 (2) K
0.20 × 0.20 × 0.16 mm

#### Data collection

Bruker Kappa APEXII diffractometer
Absorption correction: multi-scan (*SADABS*; Sheldrick, 1996 ▶) *T*_min_ = 0.593, *T*_max_ = 0.692
25160 measured reflections
6169 independent reflections
4163 reflections with *I* > 2σ(*I*)
*R*_int_ = 0.027

#### Refinement

*R*[*F* ^2^ > 2σ(*F* ^2^)] = 0.044
*wR*(*F* ^2^) = 0.127
*S* = 1.03
6169 reflections
254 parameters
3 restraints
H-atom parameters constrained
Δρ_max_ = 0.68 e Å^−3^
Δρ_min_ = −0.89 e Å^−3^

### 

Data collection: *APEX2* (Bruker, 2004 ▶); cell refinement: *APEX2*; data reduction: *APEX2*; program(s) used to solve structure: *SHELXS97* (Sheldrick, 2008 ▶); program(s) used to refine structure: *SHELXL97* (Sheldrick, 2008 ▶); molecular graphics: *PLATON* (Spek, 2003 ▶); software used to prepare material for publication: *SHELXL97*.

## Supplementary Material

Crystal structure: contains datablocks I, global. DOI: 10.1107/S1600536808007319/rk2082sup1.cif

Structure factors: contains datablocks I. DOI: 10.1107/S1600536808007319/rk2082Isup2.hkl

Additional supplementary materials:  crystallographic information; 3D view; checkCIF report

## Figures and Tables

**Table 1:** Hydrogen-bond geometry (Å, °)

*D*—H⋯*A*	*D*—H	H⋯*A*	*D*⋯*A*	*D*—H⋯*A*
C12—H12⋯O1^i^	0.93	2.56	3.472 (3)	165
C2—H2⋯O5^ii^	0.93	2.60	3.235 (3)	126
C6—H6⋯O2	0.93	2.54	2.908 (4)	104
C10—H10⋯O4	0.93	2.37	2.892 (3)	116
C13—H13⋯O1	0.93	2.28	2.863 (3)	120
C15—H15*A*⋯O5	0.97	2.31	2.911 (4)	119
C15—H15*D*⋯O2	0.97	2.16	2.895 (4)	131

Symmetry codes: (i) 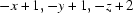; (ii) 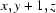.

**Fig. 1.**
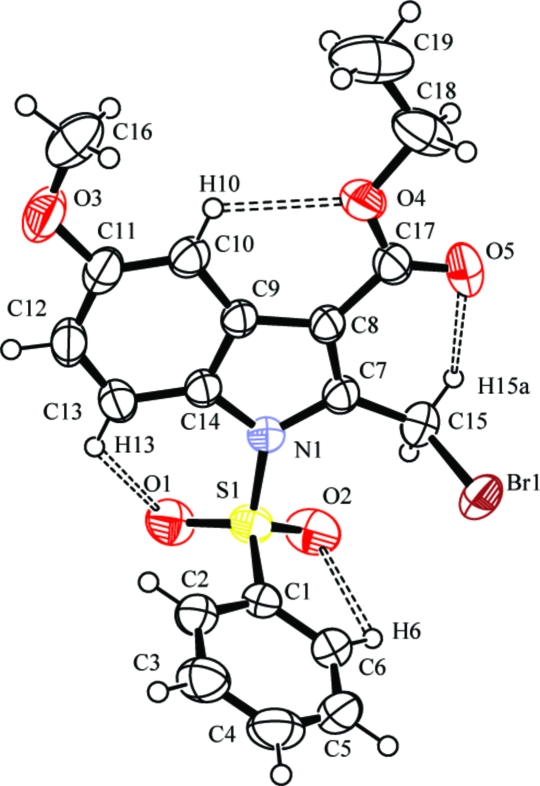
The molecular structure of I, with atom labeling scheme. Displacement ellipsoids are drawn at 50% probability level. H atoms are presented as a small spheres of arbitrary radius. Only major fragment for disordered Br1 and C15 are drawn. Intramolecular H–bonds are shown as dashed lines.

**Fig. 2.**
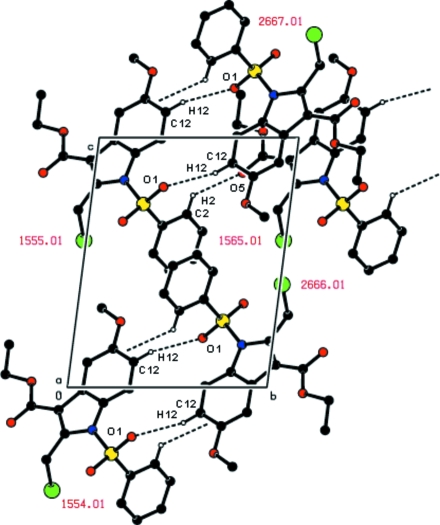
The packing of I, viewed down the a axis. Intermolecular H–bonds are shown as dashed lines H atoms not involving hydrogen bonding have been omitted for clarity. [Symmetry codes: (i) -x+1, -y+1, -z+2; (ii) x, y+1, z].

## supplementary crystallographic information

### Comment

In continuation of our studies of benzenesulfonamide derivatives, which are known to exhibit anti–bacterial (Nieto *et al.*, 2005) anti–tumour (Yang *et al.*, 2002) activities, we report the crystal structure of the title compound (I). The geometric parameters of the molecule of I (Fig. 1) agree well with the reported structures (Chakkaravarthi *et al.*, 2007; Chakkaravarthi *et al.*, 2008).

The plane of the phenyl ring forms a dihedral angle of 76.99 (6)° with the indole ring system. The N1—S1—C1 plane is orthogonal to indole ring (dihedral angle 88.70 (7)°) and makes 75.97 (9)° with the phenyl ring. The plane of indole ring is almost coplanar (dihedral angle 2.66 (7)°) with the ester group and makes 6.33 (18)° with the methoxy group.

The torsion angles O2–S1–N1–C7 and O1–S1–N1–C14 [-30.0 (2)° and 27.3 (2)°, respectively] indicate *syn*–conformation of the sulfonyl moiety. The Br1 atom is disordered over two positions with the site occupancy factors of 0.833 (14) and 0.167 (14). The molecular packing is stabilized by weak intramolecular C—H···O interactions and the crystal packing of I (Fig. 2) is stabilized by weak intermolecular C—H···O interactions (see Table).

### Experimental

Ethyl 2-(methyl)-5-methoxy-1-(phenylsulfonyl)-1*H*-indole-3-carboxylate (1g, 2.2 mmol), *N*–bromo succinimide (0.4 g, 2.3 mmol), azo–bis–isobutyronitrile (50 mg) were dissolved in 50 ml of carbon tetrachloride. Refluxed on a waterbath for 2hr. Cooled to room temperature. Succinimide was filtered off over sodium sulfate. Filtrate was evaporated under reduced pressure. Product was recrystallized from methanol. Yield: 78%.

### Refinement

The H atoms were positioned geometrically and refined using riding model with C—H = 0.93 Å and *U*_iso_(H) = 1.2Ueq(C) for aromatic  C—H, C—H = 0.97 Å and *U*_iso_(H) = 1.2Ueq(C) for CH_2_ and C—H = 0.96 Å and *U*_iso_(H) = 1.5Ueq(C) for CH_3_. The site occupancy factors for disordered Br atom is refined as Br1= 0.833 (14) and Br1A = 0.167 (14) during anisotropic refinement. The C15—Br1A distance was restrained to 1.91 (10) Å. The anisotropic displacement parameters of Br1 and Br1A were set equal by the command EADP and the anisotropic thermal parameters of C4, C5, C15 and Br1A atoms were restrained with DELU in the final cycles of refinement (Sheldrick, 2008).

### Crystal data

**Table:**

C_19_H_18_BrNO_5_S	*Z* = 2
*M**_r_* = 452.31	*F*_000_ = 460
Triclinic, *P*1	*D*_x_ = 1.578 Mg m^−^^3^
Hall symbol: -P 1	Mo *K*α radiation λ = 0.71073 Å
*a* = 8.9988 (3) Å	Cell parameters from 8207 reflections
*b* = 9.2343 (2) Å	θ = 2.2–27.5º
*c* = 11.6068 (3) Å	µ = 2.30 mm^−^^1^
α = 82.524 (1)º	*T* = 295 (2) K
β = 87.666 (2)º	Block, colourless
γ = 84.942 (3)º	0.20 × 0.20 × 0.16 mm
*V* = 952.16 (5) Å^3^	

### Data collection

**Table:**

Bruker Kappa APEXII diffractometer	6169 independent reflections
Radiation source: Fine–focus sealed tube	4163 reflections with *I* > 2σ(*I*)
Monochromator: Graphite	*R*_int_ = 0.027
*T* = 295(2) K	θ_max_ = 31.2º
ω and φ scans	θ_min_ = 1.8º
Absorption correction: multi-scan(SADABS; Sheldrick, 1996)	*h* = −13→13
*T*_min_ = 0.593, *T*_max_ = 0.692	*k* = −13→13
25160 measured reflections	*l* = −16→16

### Refinement

**Table:**

Refinement on *F*^2^	Secondary atom site location: Difmap
Least-squares matrix: Full	Hydrogen site location: Geom
*R*[*F*^2^ > 2σ(*F*^2^)] = 0.044	H-atom parameters constrained
*wR*(*F*^2^) = 0.127	*w* = 1/[σ^2^(*F*_o_^2^) + (0.054*P*)^2^ + 0.4811*P*] where *P* = (*F*_o_^2^ + 2*F*_c_^2^)/3
*S* = 1.04	(Δ/σ)_max_ < 0.001
6169 reflections	Δρ_max_ = 0.68 e Å^−^^3^
254 parameters	Δρ_min_ = −0.89 e Å^−^^3^
3 restraints	Extinction correction: None
Primary atom site location: Direct	

### Fractional atomic coordinates and isotropic or equivalent isotropic displacement parameters (Å^2^)

**Table:**

	*x*	*y*	*z*	*U*_iso_*/*U*_eq_	Occ. (<1)
Br1	0.2859 (3)	−0.0105 (2)	0.5880 (3)	0.0695 (4)	0.833 (14)
Br1A	0.2613 (8)	0.0063 (7)	0.6105 (7)	0.0695 (4)	0.167 (14)
S1	0.53695 (6)	0.26331 (6)	0.73409 (5)	0.04656 (14)	
O1	0.6066 (2)	0.3534 (2)	0.80232 (18)	0.0623 (5)	
O2	0.6249 (2)	0.1714 (2)	0.66452 (19)	0.0688 (6)	
O3	0.1927 (3)	0.2237 (2)	1.26357 (17)	0.0714 (6)	
O4	0.1493 (2)	−0.18350 (18)	1.02343 (16)	0.0551 (4)	
O5	0.2440 (3)	−0.2589 (2)	0.8592 (2)	0.0783 (6)	
N1	0.4384 (2)	0.15286 (19)	0.82973 (15)	0.0410 (4)	
C1	0.4041 (2)	0.3722 (2)	0.64784 (18)	0.0407 (4)	
C2	0.3253 (3)	0.4881 (3)	0.6931 (2)	0.0499 (5)	
H2	0.3405	0.5062	0.7686	0.060*	
C3	0.2243 (3)	0.5759 (3)	0.6245 (3)	0.0627 (7)	
H3	0.1703	0.6545	0.6537	0.075*	
C4	0.2020 (4)	0.5490 (3)	0.5135 (3)	0.0690 (7)	
H4	0.1321	0.6085	0.4682	0.083*	
C5	0.2818 (4)	0.4352 (3)	0.4690 (2)	0.0693 (7)	
H5	0.2663	0.4178	0.3935	0.083*	
C6	0.3860 (3)	0.3453 (3)	0.5360 (2)	0.0561 (6)	
H6	0.4422	0.2688	0.5058	0.067*	
C7	0.3878 (2)	0.0171 (2)	0.81460 (18)	0.0412 (4)	
C8	0.3026 (2)	−0.0297 (2)	0.90984 (18)	0.0393 (4)	
C9	0.2973 (2)	0.0781 (2)	0.98934 (17)	0.0380 (4)	
C10	0.2300 (3)	0.0839 (3)	1.10015 (19)	0.0452 (5)	
H10	0.1742	0.0093	1.1353	0.054*	
C11	0.2498 (3)	0.2044 (3)	1.1549 (2)	0.0510 (5)	
C12	0.3333 (3)	0.3160 (3)	1.1024 (2)	0.0555 (6)	
H12	0.3444	0.3958	1.1417	0.067*	
C13	0.3993 (3)	0.3117 (3)	0.9951 (2)	0.0516 (5)	
H13	0.4547	0.3871	0.9607	0.062*	
C14	0.3808 (2)	0.1902 (2)	0.93842 (18)	0.0399 (4)	
C15	0.4242 (3)	−0.0591 (3)	0.7108 (2)	0.0560 (5)	
H15A	0.4287	−0.1640	0.7346	0.067*	0.833 (14)
H15B	0.5226	−0.0356	0.6808	0.067*	0.833 (14)
H15C	0.4302	−0.1646	0.7312	0.067*	0.167 (14)
H15D	0.5176	−0.0306	0.6736	0.067*	0.167 (14)
C16	0.0958 (4)	0.1221 (4)	1.3175 (3)	0.0719 (8)	
H16A	0.0109	0.1221	1.2700	0.108*	
H16B	0.0630	0.1487	1.3922	0.108*	
H16C	0.1474	0.0259	1.3271	0.108*	
C17	0.2311 (3)	−0.1686 (2)	0.9262 (2)	0.0470 (5)	
C18	0.0781 (3)	−0.3188 (3)	1.0520 (3)	0.0738 (9)	
H18A	0.1507	−0.4023	1.0488	0.089*	
H18B	0.0008	−0.3243	0.9972	0.089*	
C19	0.0120 (4)	−0.3191 (5)	1.1716 (4)	0.1037 (15)	
H19A	0.0896	−0.3150	1.2252	0.156*	
H19B	−0.0375	−0.4070	1.1930	0.156*	
H19C	−0.0589	−0.2354	1.1739	0.156*	

### Atomic displacement parameters (Å^2^)

**Table:**

	*U*^11^	*U*^22^	*U*^33^	*U*^12^	*U*^13^	*U*^23^
Br1	0.0960 (5)	0.0791 (4)	0.0381 (5)	−0.0300 (4)	−0.0142 (5)	−0.0064 (4)
Br1A	0.0960 (5)	0.0791 (4)	0.0381 (5)	−0.0300 (4)	−0.0142 (5)	−0.0064 (4)
S1	0.0417 (3)	0.0457 (3)	0.0503 (3)	−0.0032 (2)	0.0003 (2)	0.0003 (2)
O1	0.0509 (10)	0.0655 (11)	0.0719 (12)	−0.0198 (8)	−0.0153 (8)	0.0002 (9)
O2	0.0616 (11)	0.0617 (11)	0.0763 (13)	0.0099 (9)	0.0226 (10)	−0.0013 (10)
O3	0.0890 (15)	0.0806 (14)	0.0485 (10)	−0.0023 (11)	0.0037 (10)	−0.0285 (10)
O4	0.0634 (10)	0.0476 (9)	0.0544 (10)	−0.0169 (8)	−0.0054 (8)	0.0019 (7)
O5	0.1144 (18)	0.0565 (11)	0.0729 (13)	−0.0309 (11)	0.0018 (12)	−0.0271 (10)
N1	0.0493 (10)	0.0366 (8)	0.0368 (9)	−0.0027 (7)	−0.0047 (7)	−0.0028 (7)
C1	0.0475 (11)	0.0364 (10)	0.0378 (10)	−0.0087 (8)	−0.0007 (8)	0.0004 (8)
C2	0.0592 (14)	0.0448 (12)	0.0446 (12)	−0.0003 (10)	−0.0010 (10)	−0.0046 (9)
C3	0.0667 (17)	0.0526 (14)	0.0643 (17)	0.0067 (12)	−0.0043 (13)	0.0026 (12)
C4	0.0731 (18)	0.0632 (15)	0.0666 (17)	−0.0116 (12)	−0.0220 (14)	0.0176 (12)
C5	0.100 (2)	0.0681 (16)	0.0423 (13)	−0.0282 (13)	−0.0172 (13)	0.0013 (11)
C6	0.0832 (18)	0.0457 (12)	0.0405 (12)	−0.0136 (12)	0.0018 (12)	−0.0054 (9)
C7	0.0482 (11)	0.0377 (10)	0.0378 (10)	0.0006 (8)	−0.0085 (9)	−0.0055 (8)
C8	0.0470 (11)	0.0349 (9)	0.0367 (10)	−0.0014 (8)	−0.0108 (8)	−0.0048 (7)
C9	0.0435 (10)	0.0352 (9)	0.0349 (9)	0.0020 (8)	−0.0096 (8)	−0.0046 (7)
C10	0.0501 (12)	0.0476 (12)	0.0379 (10)	−0.0016 (9)	−0.0074 (9)	−0.0051 (9)
C11	0.0588 (14)	0.0557 (13)	0.0393 (11)	0.0059 (11)	−0.0081 (10)	−0.0143 (10)
C12	0.0738 (17)	0.0448 (12)	0.0515 (13)	−0.0005 (11)	−0.0141 (12)	−0.0183 (10)
C13	0.0666 (15)	0.0386 (11)	0.0517 (13)	−0.0078 (10)	−0.0130 (11)	−0.0074 (9)
C14	0.0467 (11)	0.0357 (10)	0.0373 (10)	0.0005 (8)	−0.0099 (8)	−0.0045 (8)
C15	0.0730 (15)	0.0533 (14)	0.0440 (12)	−0.0058 (11)	0.0002 (9)	−0.0152 (10)
C16	0.0666 (18)	0.097 (2)	0.0486 (15)	0.0153 (16)	0.0053 (13)	−0.0143 (15)
C17	0.0561 (13)	0.0395 (11)	0.0464 (12)	−0.0060 (9)	−0.0160 (10)	−0.0033 (9)
C18	0.0631 (16)	0.0593 (16)	0.096 (2)	−0.0238 (13)	−0.0233 (16)	0.0205 (15)
C19	0.065 (2)	0.128 (3)	0.105 (3)	−0.030 (2)	−0.0010 (19)	0.048 (3)

### Geometric parameters (Å, °)

**Table:**

Br1—C15	1.914 (3)	C7—C15	1.484 (3)
Br1A—C15	1.9152 (10)	C8—C9	1.439 (3)
S1—O2	1.417 (2)	C8—C17	1.472 (3)
S1—O1	1.419 (2)	C9—C14	1.389 (3)
S1—N1	1.6862 (19)	C9—C10	1.405 (3)
S1—C1	1.751 (2)	C10—C11	1.379 (3)
O3—C11	1.370 (3)	C10—H10	0.9300
O3—C16	1.407 (4)	C11—C12	1.390 (4)
O4—C17	1.320 (3)	C12—C13	1.362 (4)
O4—C18	1.448 (3)	C12—H12	0.9300
O5—C17	1.208 (3)	C13—C14	1.398 (3)
N1—C7	1.405 (3)	C13—H13	0.9300
N1—C14	1.417 (3)	C15—H15A	0.9700
C1—C6	1.372 (3)	C15—H15B	0.9700
C1—C2	1.381 (3)	C15—H15C	0.9700
C2—C3	1.371 (4)	C15—H15D	0.9700
C2—H2	0.9300	C16—H16A	0.9600
C3—C4	1.369 (4)	C16—H16B	0.9600
C3—H3	0.9300	C16—H16C	0.9600
C4—C5	1.365 (5)	C18—C19	1.487 (5)
C4—H4	0.9300	C18—H18A	0.9700
C5—C6	1.388 (4)	C18—H18B	0.9700
C5—H5	0.9300	C19—H19A	0.9600
C6—H6	0.9300	C19—H19B	0.9600
C7—C8	1.364 (3)	C19—H19C	0.9600
			
O2—S1—O1	120.09 (13)	C11—C12—H12	119.1
O2—S1—N1	106.72 (11)	C12—C13—C14	117.7 (2)
O1—S1—N1	105.29 (11)	C12—C13—H13	121.1
O2—S1—C1	109.30 (12)	C14—C13—H13	121.1
O1—S1—C1	108.93 (11)	C9—C14—C13	121.2 (2)
N1—S1—C1	105.49 (10)	C9—C14—N1	107.98 (17)
C11—O3—C16	117.9 (2)	C13—C14—N1	130.8 (2)
C17—O4—C18	117.3 (2)	C7—C15—Br1	114.7 (2)
C7—N1—C14	107.77 (17)	C7—C15—Br1A	103.7 (4)
C7—N1—S1	127.88 (15)	C7—C15—H15A	108.6
C14—N1—S1	124.15 (15)	Br1—C15—H15A	108.6
C6—C1—C2	121.7 (2)	Br1A—C15—H15A	111.8
C6—C1—S1	119.67 (19)	C7—C15—H15B	108.6
C2—C1—S1	118.53 (18)	Br1—C15—H15B	108.6
C3—C2—C1	118.6 (2)	Br1A—C15—H15B	116.3
C3—C2—H2	120.7	H15A—C15—H15B	107.6
C1—C2—H2	120.7	C7—C15—H15C	111.1
C4—C3—C2	120.6 (3)	Br1—C15—H15C	107.3
C4—C3—H3	119.7	Br1A—C15—H15C	111.0
C2—C3—H3	119.7	H15B—C15—H15C	106.2
C5—C4—C3	120.4 (3)	C7—C15—H15D	111.1
C5—C4—H4	119.8	Br1—C15—H15D	102.7
C3—C4—H4	119.8	Br1A—C15—H15D	110.4
C4—C5—C6	120.3 (3)	H15A—C15—H15D	111.1
C4—C5—H5	119.8	H15C—C15—H15D	109.5
C6—C5—H5	119.8	O3—C16—H16A	109.5
C1—C6—C5	118.4 (3)	O3—C16—H16B	109.5
C1—C6—H6	120.8	H16A—C16—H16B	109.5
C5—C6—H6	120.8	O3—C16—H16C	109.5
C8—C7—N1	108.66 (18)	H16A—C16—H16C	109.5
C8—C7—C15	127.3 (2)	H16B—C16—H16C	109.5
N1—C7—C15	124.1 (2)	O5—C17—O4	123.3 (2)
C7—C8—C9	108.47 (18)	O5—C17—C8	124.7 (2)
C7—C8—C17	124.5 (2)	O4—C17—C8	112.02 (19)
C9—C8—C17	127.0 (2)	O4—C18—C19	107.3 (3)
C14—C9—C10	120.41 (19)	O4—C18—H18A	110.3
C14—C9—C8	107.12 (18)	C19—C18—H18A	110.3
C10—C9—C8	132.5 (2)	O4—C18—H18B	110.3
C11—C10—C9	117.5 (2)	C19—C18—H18B	110.3
C11—C10—H10	121.2	H18A—C18—H18B	108.5
C9—C10—H10	121.2	C18—C19—H19A	109.5
O3—C11—C10	123.9 (2)	C18—C19—H19B	109.5
O3—C11—C12	114.8 (2)	H19A—C19—H19B	109.5
C10—C11—C12	121.3 (2)	C18—C19—H19C	109.5
C13—C12—C11	121.8 (2)	H19A—C19—H19C	109.5
C13—C12—H12	119.1	H19B—C19—H19C	109.5
			
O2—S1—N1—C7	−30.0 (2)	C17—C8—C9—C10	1.3 (4)
O1—S1—N1—C7	−158.62 (19)	C14—C9—C10—C11	0.3 (3)
C1—S1—N1—C7	86.2 (2)	C8—C9—C10—C11	178.5 (2)
O2—S1—N1—C14	155.94 (18)	C16—O3—C11—C10	−6.6 (4)
O1—S1—N1—C14	27.3 (2)	C16—O3—C11—C12	174.3 (2)
C1—S1—N1—C14	−87.87 (18)	C9—C10—C11—O3	−179.0 (2)
O2—S1—C1—C6	8.6 (2)	C9—C10—C11—C12	0.0 (3)
O1—S1—C1—C6	141.6 (2)	O3—C11—C12—C13	179.0 (2)
N1—S1—C1—C6	−105.8 (2)	C10—C11—C12—C13	−0.1 (4)
O2—S1—C1—C2	−168.24 (19)	C11—C12—C13—C14	−0.1 (4)
O1—S1—C1—C2	−35.3 (2)	C10—C9—C14—C13	−0.5 (3)
N1—S1—C1—C2	77.3 (2)	C8—C9—C14—C13	−179.1 (2)
C6—C1—C2—C3	1.4 (4)	C10—C9—C14—N1	178.12 (19)
S1—C1—C2—C3	178.2 (2)	C8—C9—C14—N1	−0.5 (2)
C1—C2—C3—C4	0.1 (4)	C12—C13—C14—C9	0.4 (3)
C2—C3—C4—C5	−0.9 (5)	C12—C13—C14—N1	−177.8 (2)
C3—C4—C5—C6	0.2 (5)	C7—N1—C14—C9	0.6 (2)
C2—C1—C6—C5	−2.1 (4)	S1—N1—C14—C9	175.69 (14)
S1—C1—C6—C5	−178.8 (2)	C7—N1—C14—C13	179.0 (2)
C4—C5—C6—C1	1.2 (4)	S1—N1—C14—C13	−5.9 (3)
C14—N1—C7—C8	−0.4 (2)	C8—C7—C15—Br1	90.2 (3)
S1—N1—C7—C8	−175.24 (15)	N1—C7—C15—Br1	−90.1 (2)
C14—N1—C7—C15	179.9 (2)	C8—C7—C15—Br1A	87.5 (3)
S1—N1—C7—C15	5.0 (3)	N1—C7—C15—Br1A	−92.8 (3)
N1—C7—C8—C9	0.0 (2)	C18—O4—C17—O5	2.9 (4)
C15—C7—C8—C9	179.8 (2)	C18—O4—C17—C8	−177.4 (2)
N1—C7—C8—C17	−179.39 (19)	C7—C8—C17—O5	2.7 (4)
C15—C7—C8—C17	0.4 (4)	C9—C8—C17—O5	−176.6 (2)
C7—C8—C9—C14	0.3 (2)	C7—C8—C17—O4	−176.9 (2)
C17—C8—C9—C14	179.7 (2)	C9—C8—C17—O4	3.8 (3)
C7—C8—C9—C10	−178.1 (2)	C17—O4—C18—C19	171.9 (2)

### Hydrogen-bond geometry (Å, °)

**Table:**

*D*—H···*A*	*D*—H	H···*A*	*D*···*A*	*D*—H···*A*
C12—H12···O1^i^	0.93	2.56	3.472 (3)	165
C2—H2···O5^ii^	0.93	2.60	3.235 (3)	126
C6—H6···O2	0.93	2.54	2.908 (4)	104
C10—H10···O4	0.93	2.37	2.892 (3)	116
C13—H13···O1	0.93	2.28	2.863 (3)	120
C15—H15A···O5	0.97	2.31	2.911 (4)	119
C15—H15D···O2	0.97	2.16	2.895 (4)	131

Symmetry codes: (i) −*x*+1, −*y*+1, −*z*+2; (ii) *x*, *y*+1, *z*.
